# Burden of malaria, impact of interventions and climate variability in Western Ethiopia: an area with large irrigation based farming

**DOI:** 10.1186/s12889-022-12571-9

**Published:** 2022-01-29

**Authors:** Werissaw Haileselassie, Daniel M. Parker, Behailu Taye, Randy E. David, Endalew Zemene, Ming-Chieh Lee, Daibin Zhong, Guofa Zhou, Tesfahun Alemu, Getnet Tadele, James W. Kazura, Cristian Koepfli, Wakgari Deressa, Delenasaw Yewhalaw, Guiyun Yan

**Affiliations:** 1 School of Public Health, College of Health Sciences, Addis Ababa University, Addis Ababa, Ethiopia; 2 Population Health and Disease Prevention, College of Health Sciences, University of California at Irvine, CA 92697 Irvine, USA; 3 Department of Biology, Faculty of Natural and Computational Science, Mettu University, Mettu, Ethiopia; 4 School of Medical Laboratory Sciences, Institute of Health, Jimma University, Jimma, Ethiopia; 5 Gambella Regional Meteorology Service Center, Gambella, Ethiopia; 6 Malaria Prevention and Control Unit, Abobo District Health Office, Abobo, Gambella, Ethiopia; 7 Center for Global Health and Disease, Case Western Reserve University, Cleveland, OH 44106 USA; 8 Department of Biological Sciences 319 Galvin Life Sciences, Eck Institute for Global Health, University of Notre Dame, Notre Dame, USA; 9 Tropical and Infectious Diseases Research Centre, Jimma University, Jimma, Ethiopia

**Keywords:** Land use change, Malaria, Prevention Interventions, Ethiopia

## Abstract

**Background:**

Land use change has increasingly been expanding throughout the world in the past decades. It can have profound effects on the spatial and temporal distribution of vector borne diseases like malaria through ecological and habitat change. Understanding malaria disease occurrence and the impact of prevention interventions under this intense environmental modification is important for effective and efficient malaria control strategy.

**Methods:**

A descriptive ecological study was conducted by reviewing health service records at Abobo district health office. The records were reviewed to extract data on malaria morbidity, mortality, and prevention and control methods. Moreover, Meteorological data were obtained from Gambella region Meteorology Service Center and National Meteorology Authority head office. Univariate, bivariate and multivariate analysis techniques were used to analyze the data.

**Results:**

For the twelve-year time period, the mean annual total malaria case count in the district was 7369.58. The peak monthly malaria incidence was about 57 cases per 1000 people. Only in 2009 and 2015 that zero death due to malaria was recorded over the past 12 years. Fluctuating pattern of impatient malaria cases occurrence was seen over the past twelve years with an average number of 225.5 inpatient cases. The data showed that there is a high burden of malaria in the district. *Plasmodium falciparum* (Pf) was a predominant parasite species in the district with the maximum percentage of about 90. There was no statistically significant association between season and total malaria case number (F_3,8_: 1.982, P:0.195). However, the inter-annual total case count difference was statistically significant (F_11,132_: 36.305, *p* < 0001). Total malaria case count had shown two months lagged carry on effect. Moreover, 3 months lagged humidity had significant positive effect on total malaria cases. Malaria prevention interventions and meteorological factors showed statistically significant association with total malaria cases.

**Conclusion:**

Malaria was and will remain to be a major public health problem in the area. The social and economic impact of the disease on the local community is clearly pronounced as it is the leading cause of health facility visit and admission including the mortality associated with it. Scale up of effective interventions is quite important. Continuous monitoring of the performance of the vector control tools needs to be done.

## Background

Malaria is one of the most serious and complex public health problems in the tropical and sub-tropical parts of the world. Globally, about half of the world population (3.3 billion people) were at risk of malaria infection and around 300 to 500 million cases occurred annually, leading to approximately 1.24 million deaths each year before 2010 [1–3]. Reported malaria cases by the WHO in 34 malaria-eliminating countries decreased by 85 percent from 1.5 million in 2000 to 232,000 cases in 2010 [3]. From 2004 to 2010 deaths due to malaria decreased from 1.82million to 1.24 million [4]. Globally an estimated 219 million cases of malaria occurred in 2017, compared with 239 million cases in 2010 and 217 million cases in 2016. The reduction in malaria morbidity for the period 2015–2017 did not show significant progress though there were an estimated 20 million fewer malaria cases in 2017 compared to the number of cases in 2010[5]. In 2018, there were an estimated 228 million cases and 405,000 deaths due to malaria worldwide [6]. An estimated 229 million malaria cases occurred in 87 malaria endemic countries in 2019 with 9 million malaria case reduction compared to the number of malaria cases in 2000 [7].

Today malaria accounts for 2.6% of the total global disease burden. Africa remains the continent that has the greatest burden of malaria cases and deaths in the world. In Africa malaria is responsible for 10% of the overall disease burden [2]. In 2018, the continent accounted for 93% of malaria cases and 94% of malaria deaths [8]. Over 60% of the global cases and 90% of the global deaths occurred in sub-Saharan Africa [2]. Pregnant women and young children in Africa continue to suffer from the scourge of malaria [9]. It is also a leading cause of death among African children, causing approximately 20% of all child deaths under five years old [10].

Malaria is also a major public health problem in Ethiopia with an estimated 68% (52 million people) of the population living in malarious areas which covers 75% of the land of the country [10]. However, significant progress has been made in reducing malaria over the last two decades [11] which could be due to scale up of high impact interventions [12, 13]. In Ethiopia, the prevention and control of malaria involves early diagnosis and prompt treatment, selective vector control using indoor residual spraying (IRS), insecticide treated nets (ITNs) and environmental management [14]. Although the current malaria vector control strategies are effective in decreasing patient morbidity and mortality, malaria is still among the major public health problems in Ethiopia. It is the most frequent cause of out-patient (10—40%) and in-patient admission (13—26%) nationwide with corresponding mortality rates of 13–35% [1, 15]. It is also one of the leading causes of morbidity and mortality among adult populations [16, 17]. *P. falciparum* is the major (60%) cause of malaria disease occurrence followed by *P. vivax* with a percentage of 40 [1, 15]. In recent years, the number of *P. vivax* cases is increasing [18, 19].

Therefore understanding the spatial and temporal heterogeneity in malaria incidence is important to consolidate malaria control planning and monitoring effort through cost effective allocation of intervention resources [20]. Moreover, there are efforts to use rainfall data for malaria prediction in various parts of the world including Ethiopia since climatic conditions play an important role in malaria prediction [21].

Land use change has increasingly been expanding throughout the world in the past decades due to the need to satisfy the increasing human demand for food, fiber, water, and shelter. It has significantly altered a great portion of the earth’s surface [22]. It can have profound effects on the spatial and temporal distribution of vector borne diseases like malaria through ecological and habitat change [23–26]. In the past decade, sub-Saharan African countries have experienced a new era of large dam constructions and expansion of irrigated agricultural farms. Likewise severe droughts that have plagued Ethiopia for many years led the country to extensive diversion of water bodies for development. The purpose of this water resource development has been for initiation of rural irrigation schemes for agriculture and construction of dams for electricity to improve the socio-economic situation in the country. This greatly helped the fight against poverty there by reducing vulnerability to drought which is due to dependency on rain-fed agriculture. The Ethiopian agriculture led economic approach has primarily focused on expanding irrigation in the last decade in the effort to promote economic growth. In line with this major investments have been made in water resource development particularly in Gambella where this study was conducted. The construction of dams and irrigation has boosted rice farming in the area, but has led to environmental changes.

These environmental changes may have unforeseen ecologic consequences that adversely affect human health by aggravating malaria transmission [27]. Moreover, the water resource development activities may also increase the transmission of malaria [28]. Therefore, there is a need to assess the local malaria disease burden under this intense environmental modification. Trends in malaria disease occurrence need to be investigated so that examining the pattern before and after the land use change can easily be done. This would help assessment of the possibility of hypothesizing effects of the land use change on malaria disease occurrence in the study area. Besides, assessing the performance of local malaria control tools and the effect of climate will be of paramount importance to design effective strategies. Hence the current study was designed to assess the patterns in the occurrence of malaria and impact of local malaria prevention and control tools as well as climate in an area where there is land use change.

## Methods

### Study site

The study was conducted in Abobo district of Gambella region in Western Ethiopia. It is located 811km west of the capital Addis Ababa and 42 km from the capital of Gambella region. The study area is found at latitude 7.94 and longitude 34.03472 at an elevation ranging from 400-600 m above sea level. Major water bodies in this district include Lake Alwero. 20% of the district is covered by forest [29]. The estimated total population of the district for 2019 was 26,080. The district has a population density of 5.05 per km2 with 26% living in urban settings. Abobo experiences average daily temperature of 25 °C to 30 °C with a daily range of about 15.3 °C. The maximum temperature extends to 41.02 °C during February, March and April. The minimum average temperature (17.8 °C) occurs in December and January. The mean relative humidity is about 82% and drops down to as far as 37.4%. The total annual rainfall ranges between 827 to 1617 mm. Long rains occur between May, June, July, August, September, and October and long dry season occurs between November, December, January, February, March, and April each year. The hot and humid condition, coupled with seasonal rainfall, creates favorable environment for mosquito breeding.

**Fig. 1:**
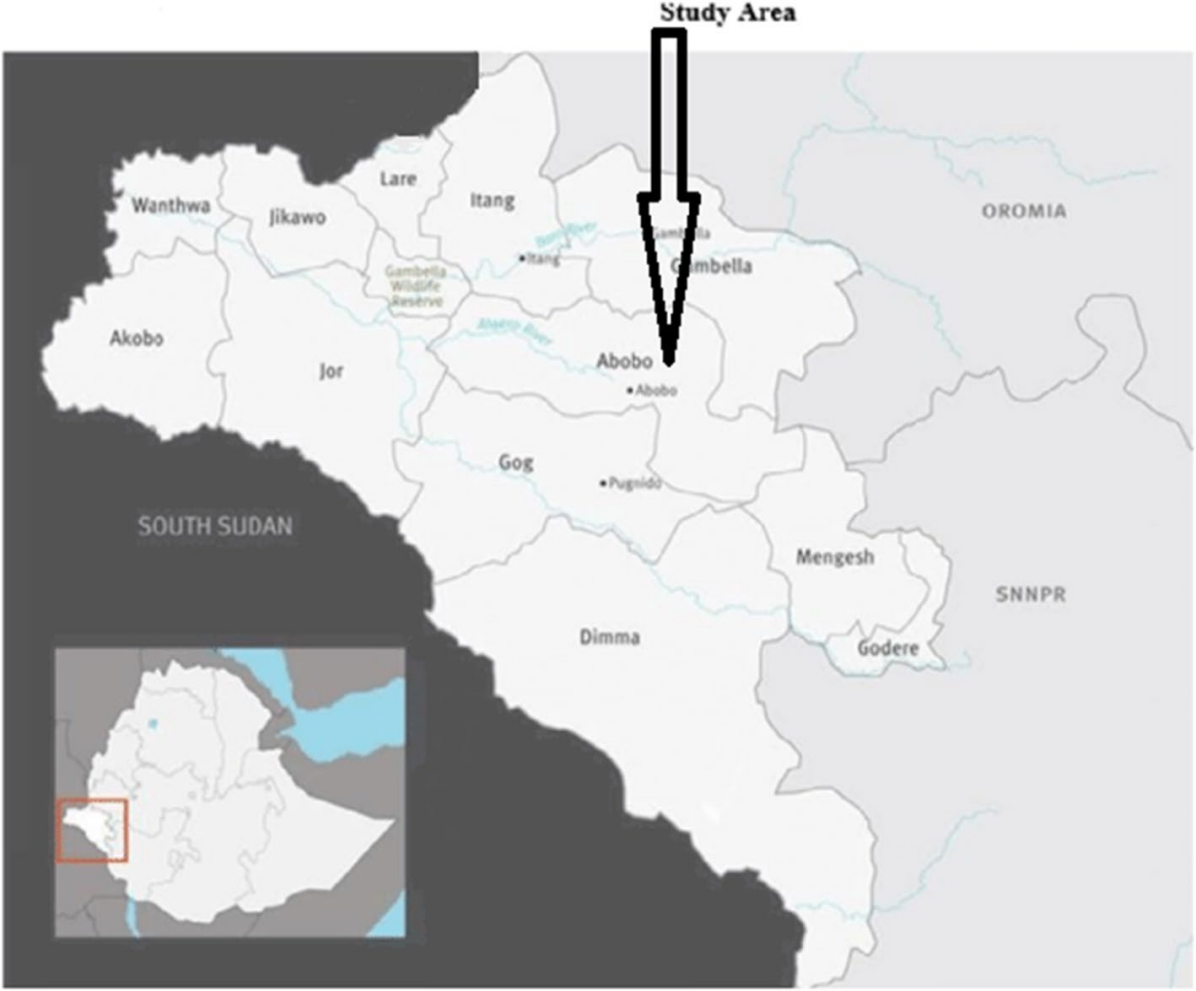
Map showing the study site (Abobo District), Gambella, Ethiopia.(The map was created by ourselves using Arc GIS 10.3 and Surfer 8 software)

The main socio-economic activities of the community are farming (cotton, maize, sorghum and fruit (mango, papaya and banana) plantations) and fishing from the Alwero dam. Alwero dam is also used for large scale irrigation of rice owned by an agricultural company. This agricultural development project has created job opportunity for more than 2000 workers. Houses are traditional type constructed of mud and wood, the majority with thatched roofs and very few with corrugated iron sheets. Generally, the district has 19 villages and four health centers. Moreover, there are 16 health posts staffed by health extension workers. According to the information we obtained from the woreda health office, generally in the study area there are very limited number (only 4) of private clinics. They are highly spatially skewed in their distribution being concentrated in Abobo town, the center of the district. In this town, Abobo Health Center is located. It is staffed by both Ethiopian and European health care providers. It serves as a referral health center in the district as it is better placed to provide comparatively better health care. The major ethnic groups in the area are Anuak, Kambata, Amhara, Oromo, Tigray and Gurage. Orthodox, Protestant, Catholic and Muslim are religions that the people in the area follow. Map of the study area is shown below in Fig. 1 [29].

### Study design

A descriptive ecological study was conducted by reviewing health service records at local health facilities and Abobo district health office. The records were reviewed to extract data on malaria morbidity, mortality, and prevention and control methods. Moreover, meteorological data (Tem, Rainfall and humidity) were obtained from Gambella region Meteorology Service Center and National Meteorology Authority (NMA) head office.

### Data collection

Retrospective malaria data between 2008 and 2019 G.C were extracted from records kept at health institutions. The data were collected by trained health professionals under the supervision of the research team. It was collected from 4 health centers and 16 health posts in the district.

Hence the study included both clinically diagnosed malaria patients as well as patients who were diagnosed by microscope and RDT. Characteristics of presumptive and confirmed malaria patients were collected. Data on malaria mortality and prevention and control methods were also collected. Besides, data on minimum temperature, maximum temperature, mean temperature, total rainfall and relative humidity of the district were obtained from Gambella regional Meteorology Service Center.

### Data analysis

Data were checked for completeness and consistency. It was entered and processed in Microsoft Excel and analyzed using SPSS version 20(SPSS INC. Chicago, II, USA). Burden of malaria was calculated as total number of confirmed malaria cases divided by the total population at risk multiplied by one thousand. Malaria positivity was calculated as number of slides/RDT positive for malaria divided by total number of slides/RDT performed for malaria multiplied by one hundred. The percentage of *P. falciparum* (Pf) cases was computed as number of confirmed Pf malaria cases divided by total number of confirmed malaria cases multiplied by one hundred. Trend of malaria cases was presented using line graphs. Moreover, ANOVA test was performed to assess the inter-annual case number and meteorological factors variation. Annual species composition was calculated for malaria parasites. Seasonality by total malaria cases was determined. The impact of climate and control measures on the annual number of malaria cases was assessed using a Poisson regression model. Model selection was done based on Akaike Information criterion (AIC). Since there might be serial correlation among observations in the time series, autocorrelation analysis was conducted. Moreover, cross correlation analysis was also done to examine correlation between time series variables. Winters’ multiplicative model was applied for forecasting total malaria cases to be observed in subsequent years.

## Results

### Trends in malaria morbidity and mortality at Abobo district

**Fig. 2:**
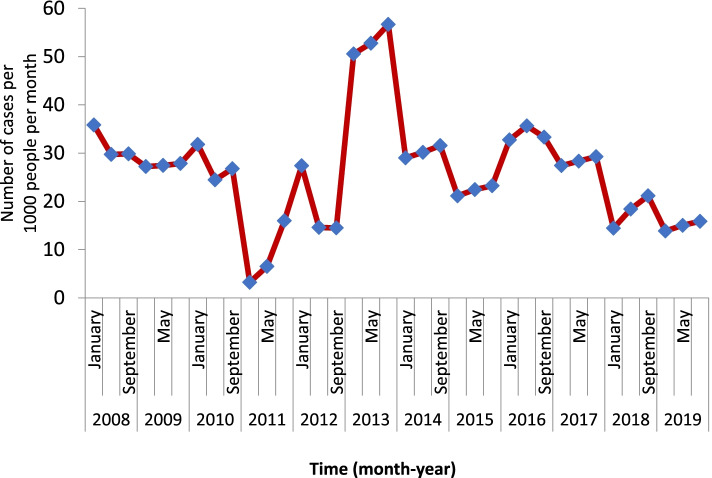
Trends of clinical malaria incidence in Abobo district, Gambella region, Ethiopia from 2008–2019

**Fig. 3:**
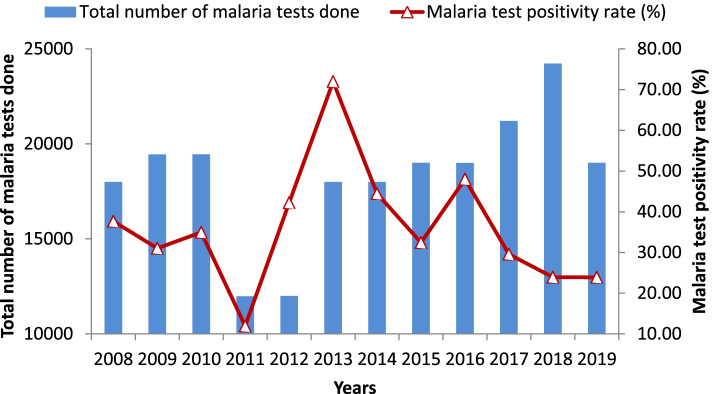
Test positivity rate in Abobo district, Gambella, Ethiopia from 2008–2019

**Fig. 4:**
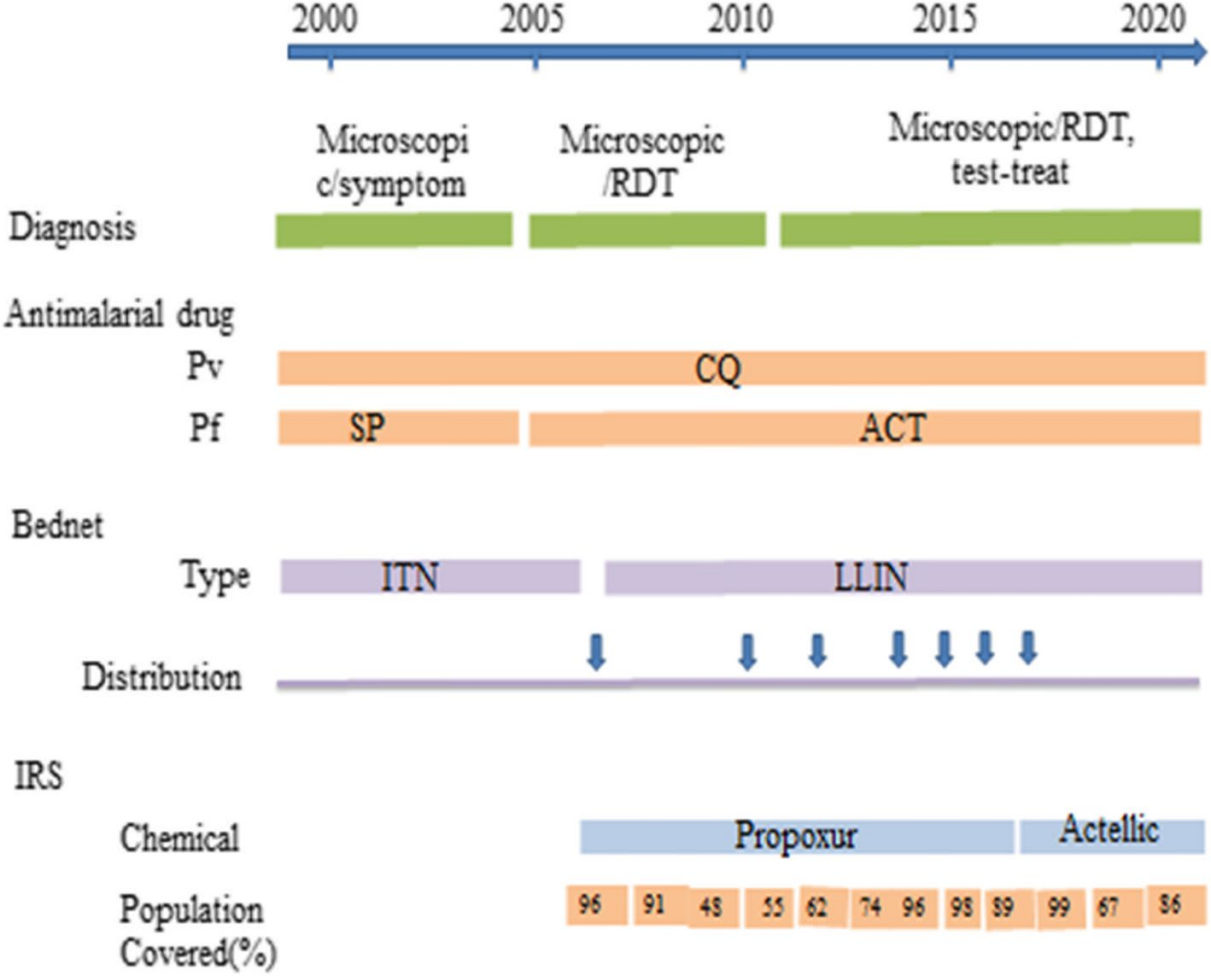
Malaria prevention, diagnosis and treatment in Abobo district, Gambella, Ethiopia from 2008–2019

The clinical malaria incidence in the last twelve years showed highly fluctuating patterns. It had sharply increased in 2013 with a sharp decline in the subsequent two years (2014 and 2015) followed by a slight increase over the next year. The peak total malaria incidence was recorded in 2013 with a monthly malaria incidence of about 57 cases per 1000 people. However, it had dropped noticeably over the past four years (Fig. 2). Yet the incidence remained high in the area. The highest malaria positivity rate was observed in 2013. Since then, it has been progressively reduced with commensurate improvement in the number of tests done (Fig. 3). Abobo district malaria related interventions over the years are shown in Fig. 4 below.

Similarly, the number of deaths due to malaria peaked in 2013 and 2014, during which six deaths each were recorded. Only in 2009 and 2015 that zero death due to malaria was recorded over the past 12 years. An alarming number of severe malaria cases were detected in 2012 followed by the number of impatient cases observed in 2017. However, noticeable overall reduction in impatient malaria cases was observed after 2012. Irregular pattern of impatient malaria cases occurrence was seen over the past twelve years with an average number of 225.5 inpatient cases.

**Fig. 5:**
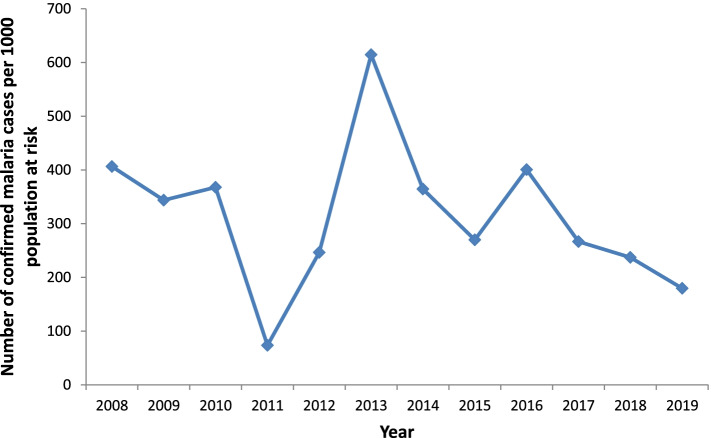
Burden of malaria over the past twelve years in Abobo district, Gambella, Ethiopia, 2008–2019

An overwhelming burden of malaria was observed in 2013. The data shows that there is a high burden of malaria in the district. The lowest burden of malaria was seen in 2011 with 73 cases per 1000 population per year. Though there was a reduction in the burden of malaria in the past three years, it was very high (Fig. 5).

### Malaria parasite species distribution

**Fig. 6:**
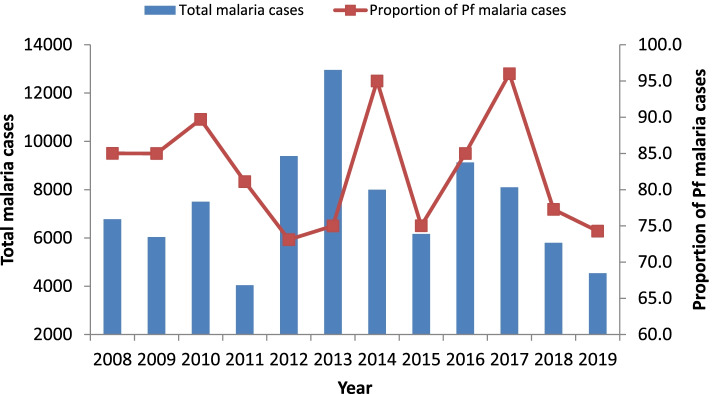
trends in malaria cases and proportion of Pf cases in Abobo district, Gambella Ethiopia, 2008–2019

*P. falciparum* was a predominant parasite species in the district over the 12 year time period with a maximum percentage of about 90. There was a fluctuating trend in the proportion of *P. falciparum* cases. A change in parasite distribution was clearly pronounced in the past two years with an increase in the number of *P. vivax* cases (Fig. 6). The maximum number of *P. vivax* cases was reported in 2012 which coincides with the booming of vast agricultural development in the area.

### Total malaria case seasonal variation and forecasting

**Fig. 7:**
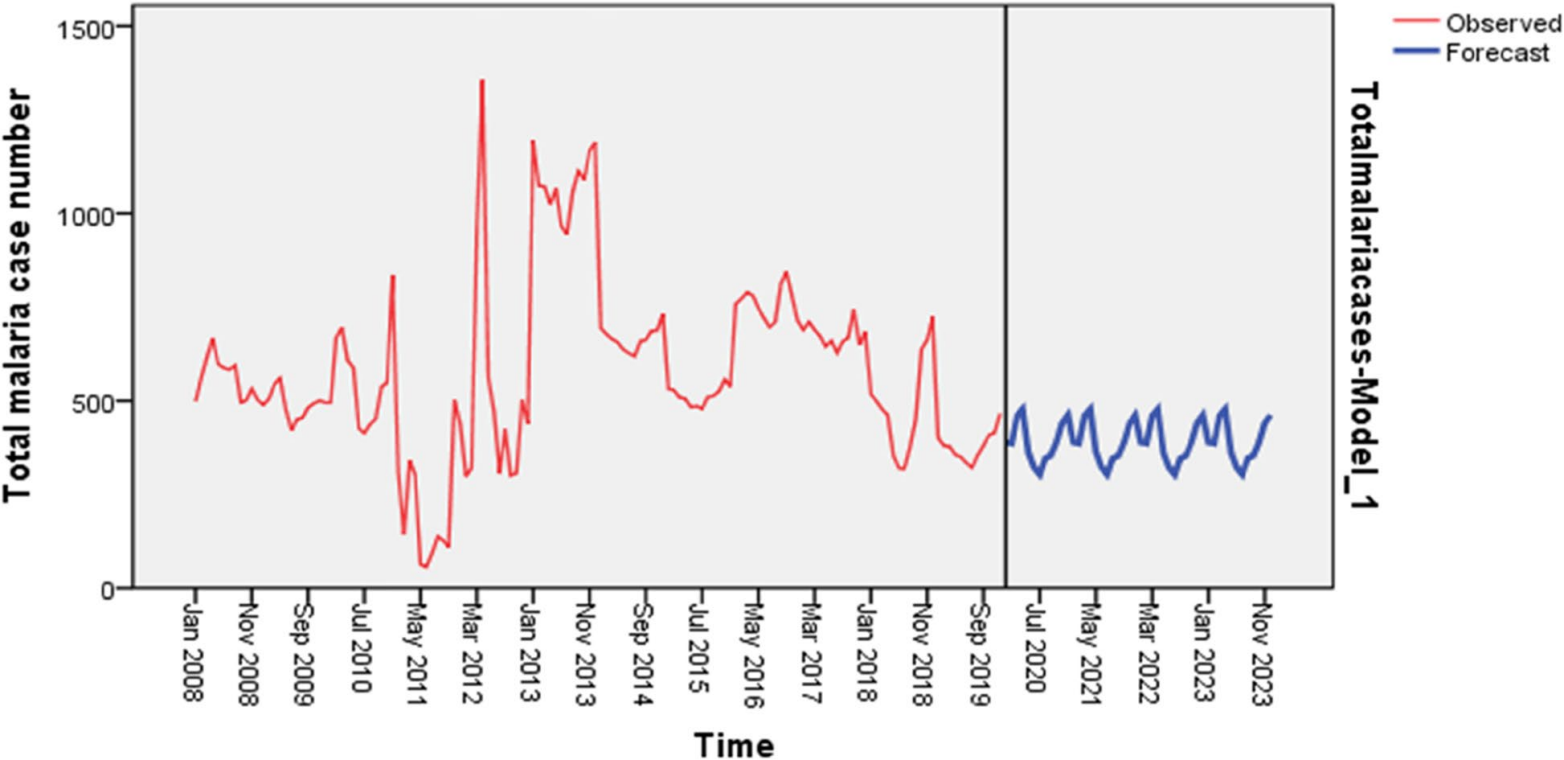
Five year forecast for malaria case count at Abobo district, Gambella, Ethiopia, 2020

Insignificant effect of season on total malaria case number was observed (F_3,8_: 1.982, P:0.195). Malaria cases occurred in the district in every month and season with slight fluctuation over months (p:0.733). High numbers of cases were occurred from May to August and from September to December. Total malaria cases forecasting was done based on the 12 years monthly malaria data for the next five years. The result showed that the total malaria case number will remain at (replicate) its current level (magnitude) over the next five year time period. The total malaria case count pattern over the past 12 years period and over the next five years period is shown in the figure below (Fig. 7). The inter annual total case count difference was highly significant (F_11,132_: 36.305, *p* < 0001).

### Trends of total malaria cases and meteorological factors in Abobo district from 1986–2019

**Fig. 8:**
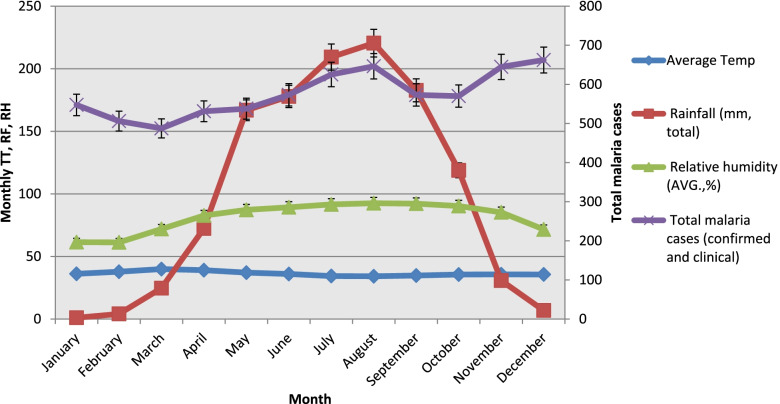
total malaria cases and meteorological factors in Abobo district from1986 -2019

**Fig. 9:**
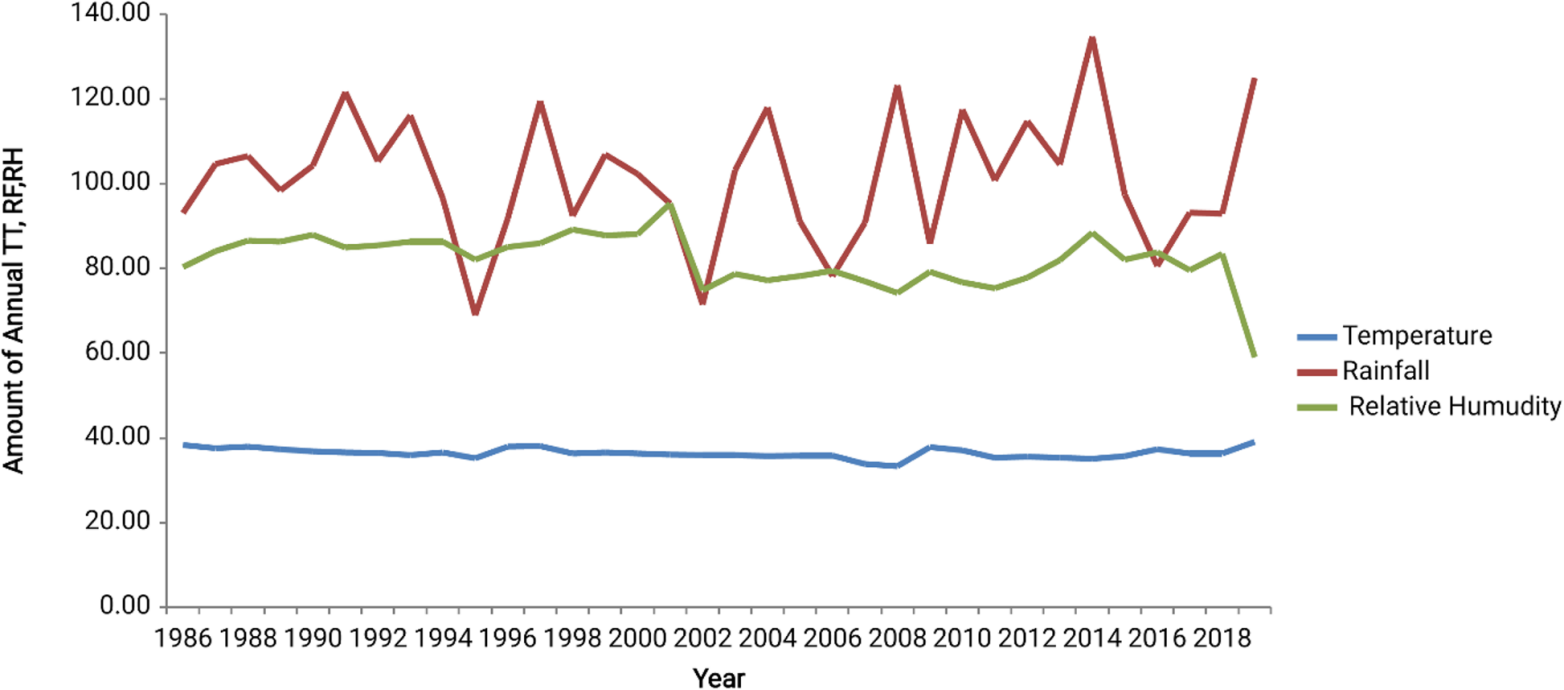
Annual meteorological factors pattern in Abobo district from 1986 -2019

As shown in Fig. 8, the climatic conditions from April to November were favorable for total malaria case count increment. Similarly, malaria case numbers peaked in December following the major rain falling over May to October. In the district, monthly mean temperature ranged from 34.14 °C to 39.9 °C. Moreover, a highly fluctuating pattern of rainfall was observed over the years from 1986 to 2019 (Fig. 9).

### The effect of climate and control measures on total malaria cases

Total malaria case count modeling was done using climatological factors and prevention performance based variables to evaluate their impact on total malaria cases over time. Accordingly, number of ITN distributed (P:0.035), unit structure sprayed (*P* < 0.001), Tmax (*P* < 0.001), Tmin (*P* < 0.001), Tmean (*P* < 0.001), rainfall (*P* < 0.001) and humidity (*P* < 0.001) were found to have significant effect on local malaria case count. Moreover, we also observed significant positive correlation between 4 months lagged precipitation and total malaria cases. Similarly, total malaria case count and rain fall were found to be strongly contemporaneously correlated. Moreover, 3 months lagged humidity had significant positive effect on total malaria cases. Total malaria case count had shown two months lagged carry on effect. Spray month was found to have insignificant effect on total malaria cases (F: 1.316, df: 5 p:0.36).

## Discussion

This study showed that overall there was high burden of malaria in the area, despite the reduction in recent years. Malaria incidence peaked in 2012 and 2013 following the development of rice production and irrigation project by an agricultural company. This was also the time of the beginning of extensive agricultural activities by other private investors. According to the district health office, it has been observed that there is extensive change of land use/landcover pattern with extensification of agriculture though no land use/cover data (spatial and temporal) is collected. This has converted pasturelands and natural vegetation to farm lands. The booming of agricultural development particularly the irrigation-based cultivation of rice in the area may result in ecological transformation leading to the creation of favorable conditions for mosquito breeding; thereby igniting the transmission of malaria. Moreover, these huge agricultural investments attracted people from the highlands to the district. As a result, the influx of people from low malaria transmission or malaria free areas had increased the pool of highly susceptible individuals for the disease as these are people with low immunity.

The reduction in the burden of malaria during the past four years may be attributed to the scale-up of effective interventions at the national and local levels. This is consistent with the national report of reduction of malaria burden throughout the country in the past few years [30]. 36% mean annual positivity rate of malaria was observed which is similar to the findings reported from Adi Arkay (36.1%) and Kola Diba (39.6%) [14, 31] but much higher than the malaria positivity rate reported from Ataye (8.4%) and Wolkite (8.56%) [32, 33]. The difference might be due to the variation in health seeking behavior, malaria prevention intervention implementation and malaria diagnosis practice and skill beside the difference in eco-epidemiological setting.

Similarly, highest number of deaths was reported in 2012 and 2013. This might be attributed to the highest number of malaria cases that occurred during the same time period. It might also be due to inadequate case management by the local health facilities due to the overwhelmingly large number of cases and delay in reporting to local health facilities by the community. The shock had resulted a high percentage of *P.falciparum* cases in the district which was followed by a large number of inpatient malaria cases due to severe malaria as high *P.falciparum* cases increases the chance of seeing severe malaria cases which likely lead to death unless prompt treatment is in place. It is only in 2009 and 2015 that zero death was recorded over the past 12 years. This is lower than the national reported number [30]. The discrepancy might be due to the difference in the scope of the study between the two studies beside the tendency of not reporting death due to malaria.

Irregular pattern of in-patient malaria cases was observed over the past twelve years. There was an overall reduction of inpatient case number. This might be due to the decline in the burden of malaria in the country as a result of scale up of high impact interventions. Moreover, the high percentages of *P. falciparum* malaria cases may have contributed to the high number of sever malaria cases that occurred in the district. This showed that malaria is one of the leading causes of admission to health facilities in the area.

Considerable change in parasite distribution was observed over the past 12 years in the district. There was an increasing trend of vivax cases. However, *P. falciparum* remained as a predominant malaria species in the area throughout the twelve years. It accounted for 73–96% of the cases. The change in parasite species distribution was seen since 2012. This high contribution of *P. falciparum* for malaria positivity in the area may increase admission and death due to malaria as it is responsible for most of the severe forms of malaria. This has its implication in resource mobilization to control malaria disease in general and prevent the occurrence of severe form of malaria. The change in parasite distribution might be due to the migration of *P. vivax* cases from other parts of the country to look for income generating labor as the area is a development corridor. The parasite distribution contradicted with the national parasite distribution [34]. This is due to the difference in scope between the two studies. Similar findings were reported from Kola Diba and Ataye, where 75% and 78.2% of the cases were due to *P. falciparum*, respectively[14, 32]. In contrast a similar study conducted at Wolkite found a high prevalence of *P. vivax* cases (69.7%) followed by *P. falciparum* (29.3%) [33].

This study showed that malaria transmission in the district is sub-perennial. Peaks of markedly greater intensity were observed following the minor and major rainy seasons. Similar findings were reported by other studies conducted in Kola Diba, Halaba, Jimma and Ataye [14, 19, 32, 35]. The forecast for the coming next five years showed that the disease will continue to put its impact on the health of local community. It will maintain its current high level of occurrence unless the high impact interventions are scaled up by taking vulnerability into consideration. The inter-annual total case count fluctuation was found to be very significant. This might be due to a very significant inter- annual variations of measured meteorological factors that was observed in this study. This indicated the need for early warning system by implementing strong surveillance.

In our modeling analysis we observed the significant effect of ITN, IRS, maximum temperature, minimum temperature, mean temperature, precipitation and humidity. The study also indicated two months lagged carry on effect of total malaria cases. Similarly, significant three months lagged effect of humidity was seen on the total malaria cases. Since this study utilized secondary data to describe the malaria situation in a district, it might have some limitations peculiar to this data source.

## Conclusion

Malaria was and will remain to be a major public health problem in the area. The social and economic impact of the disease on the local community is clearly pronounced as it is the leading cause of health facility visit and admission including the mortality associated with it. This should draw attention from malaria control program or policy makers. Scale up of effective interventions is quite important. Local malaria disease dynamics was strongly associated with climate factors despite the insignificant variation over seasons. There was abrupt rise in case number that immediately followed the development of rice production and irrigation project by an agricultural development PLC including other concomitant agricultural investment in the district. Thus, any development endeavor that may cause environmental modification should take into consideration the potential detrimental effect associated with it by applying environmental impact assessment. The sub-perennial type of transmission of malaria and high burden of the disease in the area needs due attention for evidence based strong disease control strategy and resource mobilization. Since the disease showed high seasonality, program resource mobilization should take this into consideration. Further analytical studies need to be conducted to have deep insight on the dynamics of the disease occurrence with the identification of transmission related biotic and abiotic factors that can be an input for local as well as national malaria control program so that the finding can be transferred to areas with similar eco-epidemiologic settings. This assists the disease control efforts in similar eco-epidemiologic settings.

## Data Availability

The datasets used for the current study are available from the corresponding author on reasonable request.

## Abbreviations

IRS: Indoor Residual Spray
ITNs: Insecticide Treated Nets
PLC: Private Limited Company
RDT: Rapid Diagnostic Test
NMA: National Meteorology Authority
SPSS: Statistical Package for Social Science
ANOVA: Analysis of Variance

## References

[1] World Health Organization (CH) (2008). Health impact assessment.

[2] World Health Organization (CH) (2018). Universal access to core malaria interventions in high-burden countries.

[3] World Health Organization (CH) (2011). World malaria report 2011.

[4] Cohen MJ, Smith LD, Cotter C, Ward A, Yamey G, Sabot JO, Moonen B (2012). Malaria resurgence: a systematic review and assessment of its causes. Malaria journal..

[5] World Health Organization (CH) (2018). World malaria report 2018.

[6] World Health Organization. The world malaria report 2019 at a glance: WHO; 4 Dec 2019. Available from: https://www.who.int/news-room/feature-stories/detail/world-malaria-report-2019).

[7] World Health Organization (CH) (2020). World malaria report 2020: 20 years of global progress and challenges.

[8] World Health Organization (2021). Malaria key facts.

[9] World Health Organization (CH) (2019). World malaria report 2019.

[10] World Health Organization (2006). Malaria vector control and personal protection.

[11] Ministry of Health (ET). Health sector transformation plan. Addis Ababa: MOH; 2015/16 to 2019/20. https://www.globalfinancingfacility.org/ethiopia-health-sector-transformation-plan 201516–201920 (2015) Accessed 20 June 2021.

[12] Abeku TA, Helinski ME, Kirby MJ, Kefyalew T, Awano T, Batisso E (2015). Monitoring changes in malaria epidemiology and effectiveness of interventions in Ethiopia and Uganda: beyond Garki project baseline survey. Malar J.

[13] Yimer F, Animut A, Erko B, Mamo H (2015). Past five-year trend, current prevalence and household knowledge, attitude and practice of malaria in Abeshge, south-central Ethiopia. Malar J.

[14] Alemu A, Muluye D, Mihret M, Adugna M, Gebeyaw M (2012). Ten year trend analysis of malaria prevalence in Kola Diba, North Gondar. Northwest Ethiopia Parasite Vectors.

[15] RTI (2007). Integrated Vector Management Programs for Malaria Control.

[16] Delil RK, Dileba TK, Habtu YA, Gone TF, Leta TJ (2016). Magnitude of malaria and factors among febrile cases in low transmission areas of Hadiya zone, Ethiopia: a facility based cross sectional study. PLoS One.

[17] Belete EM, Roro AB (2016). Malaria prevalence and its associated risk factors among patients attending Chichu and Wonago Health Centres, South Ethiopia. Journal of Research in Health Sciences.

[18] Gebretsadik D, Feleke G, Fiseha M (2018). Eight-year trend analysis of malaria prevalence in Kombolcha, South Wollo, north-central Ethiopia: a retrospective study. Parasites Vectors.

[19] Shamebo T, Petros B (2019). Trend analysis of malaria prevalence in Halaba special district. Southern Ethiopia BMC Res Notes.

[20] Alemu K, Worku A, Berhane Y (2013). Malaria Infection Has Spatial, Temporal, and Spatiotemporal Heterogeneity in Unstable Malaria Transmission Areas in Northwest Ethiopia. PLoS ONE..

[21] Briët JTO, Vounatsou P, Gunawardena MD, Galappaththy NLG, Amerasinghe HP (2008). Temporal correlation between malaria and rainfall in Sri Lanka. Malar J.

[22] Foley J, DeFries R, Asner GP, Barford C, Bonan G, Carpenter SR (2005). Global Consequences of Land Use. Science..

[23] Grillet M (2000). Factors associated with distribution of Anopheles aquasalis and Anopheles oswaldoi (Diptera: Culicidae) in a malarious area, northeastern Venezuela. J Med Entomol..

[24] Patz J, Graczyk T, Geller N, Vittor AY (2000). Effects of environmental change on emerging parasitic diseases. Int J Parasitol..

[25] Norris DE (2004). Mosquito-borne diseases as a consequence of land use change. EcoHealth.

[26] Poff N, Olden J, Merritt D, Pepin D (2007). Homogenization of regional river dynamics by dams and global biodiversity implications. ProcNatlAcadSci.

[27] Jaleta KT, Hill ShR, Seyoum E, Balkew M, Gebre-michael T, Ignell R (2013). Agro-ecosystems impact malaria prevalence: large-scale irrigation drives vector population in western Ethiopia. Malar J.

[28] Yewhalaw D, Legesse W, Van Bortel W, Gebre-Selassie S, Kloos H, Duchateau L (2009). Malaria and water resource development: the case of Gilgel Gibe hydroelectric dam in Ethiopia. Malaria Journal..

[29] Central Statistical Agency (2007). Atlas of the Ethiopian Rural Economy.

[30] Presidents Malaria Initiative (2018). Malaria Operational Plan FY 2019.

[31] Tesfa H, Bayih AG, Zeleke AJ (2018). A 17-year trend analysis of malaria at Adi Arkay, North Gondar zone. Northwest Ethiopia Malar J.

[32] Getacher DF, Gebretsadik D, Gebreweld A (2018). Analysis of the trend of malaria prevalence in Ataye, North Shoa, Ethiopia between 2013 and 2017. Malar J.

[33] Solomon A, Kahase D, Alemayehu M (2020). Trend of malaria prevalence in Wolkite health center: an implication towards the elimination of malaria in Ethiopia by 2030. Malar J.

[34] Ministry of Health (ET) (2016). Ethiopia National Malaria Indicator Survey 2015.

[35] Alemu A, Abebe G, Tsegaye W, Golassa L (2011). Climatic variables and malaria transmission dynamics in Jimma town. South West Ethiopia Parasit Vectors.

